# Bee Venom-Loaded Niosomes as Innovative Platforms for Cancer Treatment: Development and Therapeutical Efficacy and Safety Evaluation

**DOI:** 10.3390/ph17050572

**Published:** 2024-04-29

**Authors:** Maria Beatriz Pinto, Patrícia C. Pires, Ricardo C. Calhelha, Ana Rita Silva, Maria João Sousa, Miguel Vilas-Boas, Soraia I. Falcão, Francisco Veiga, Pooyan Makvandi, Ana Cláudia Paiva-Santos

**Affiliations:** 1 Department of Pharmaceutical Technology, Faculty of Pharmacy of the University of Coimbra, University of Coimbra, Azinhaga de Santa Comba, 3000-548 Coimbra, Portugal; 2 REQUIMTE/LAQV, Group of Pharmaceutical Technology, Faculty of Pharmacy of the University of Coimbra, University of Coimbra, 3000-548 Coimbra, Portugal; 3 Health Sciences Research Centre (CICS-UBI), University of Beira Interior, Av. Infante D. Henrique, 6200-506 Covilhã, Portugal; 4 Centro de Investigação de Montanha (CIMO), Instituto Politécnico de Bragança, Campus de Santa Apolónia, 5300-253 Bragança, Portugalmvboas@ipb.pt (M.V.-B.); sfalcao@ipb.pt (S.I.F.); 5 Laboratório Associado para a Sustentabilidade e Tecnologia em Regiões de Montanha (SusTEC), Instituto Politécnico de Bragança, Campus de Santa Apolónia, 5300-253 Bragança, Portugal; 6 The Quzhou Affiliated Hospital of Wenzhou Medical University, Quzhou People’s Hospital, Quzhou 324000, China; 7 Centre of Research Impact and Outreach, Chitkara University, Rajpura 140417, India; 8 Department of Biomaterials, Saveetha Dental College and Hospitals, SIMATS, Saveetha University, Chennai 600077, India

**Keywords:** anti-inflammatory, anticancer, antitumor, bee venom, in vitro, nanosystems, natural compounds, niosomes

## Abstract

Despite past efforts towards therapeutical innovation, cancer remains a highly incident and lethal disease, with current treatments lacking efficiency and leading to severe side effects. Hence, it is imperative to develop new, more efficient, and safer therapies. Bee venom has proven to have multiple and synergistic bioactivities, including antitumor effects. Nevertheless, some toxic effects have been associated with its administration. To tackle these issues, in this work, bee venom-loaded niosomes were developed, for cancer treatment. The vesicles had a small (150 nm) and homogeneous (polydispersity index of 0.162) particle size, and revealed good therapeutic efficacy in in vitro gastric, colorectal, breast, lung, and cervical cancer models (inhibitory concentrations between 12.37 ng/mL and 14.72 ng/mL). Additionally, they also revealed substantial anti-inflammatory activity (inhibitory concentration of 28.98 ng/mL), effects complementary to direct antitumor activity. Niosome safety was also assessed, both in vitro (skin, liver, and kidney cells) and ex vivo (hen’s egg chorioallantoic membrane), and results showed that compound encapsulation increased its safety. Hence, small, and homogeneous bee venom-loaded niosomes were successfully developed, with substantial anticancer and anti-inflammatory effects, making them potentially promising primary or adjuvant cancer therapies. Future research should focus on evaluating the potential of the developed platform in in vivo models.

## 1. Introduction

Cancer remains one of the most incident diseases globally with high mortality rates, having been estimated to affect almost 2,000,000 people just in the United States of America in the year of 2023, with more than 600,000 related deaths, and having similar incidence rates all over the world [1,2,3]. This not only leads to substantial out-of-pocket costs for patients and caregivers, due to necessary in-hospital care, medication, and medical consultations, making up to 16 to 42% of their annual income, but also relevant global economic burden, being estimated to cost up to $25.2 trillion in international dollars in 2050 [4,5,6]. Furthermore, while life-expectancy keeps increasing, age itself becomes an important risk factor for cancer development, due to the aging process bringing about several relevant biological changes linked to cancer pathogenesis, leading to a reduction of late-life quality, increased disability, and overall rise in health costs [7,8,9].

Although the fast-paced advances in medicine are today able to prolong the life of these patients, and even cure them in some cases, most common cancer therapies, such as chemotherapy, radiotherapy, immunotherapy, and surgery, are linked with poor prognosis and/or severely debilitating side effects, such as nausea and vomiting, pain, fatigue, depression, hair loss, and immune system debilitation, which can lead to opportunistic infections due to the body’s higher vulnerability and low defenses [10,11,12]. Additionally, although novel technologies have recently emerged, with recent advances including various targeted therapies, and even robotics and artificial intelligence, there is still a long way towards the effective clinical testing, and, subsequently, commercialization of these potential treatments, as well as guaranteeing that they are cost-effective [13,14,15,16,17]. Hence, there is an urgent need for new, more effective, and safer therapies.

**Figure 1:**
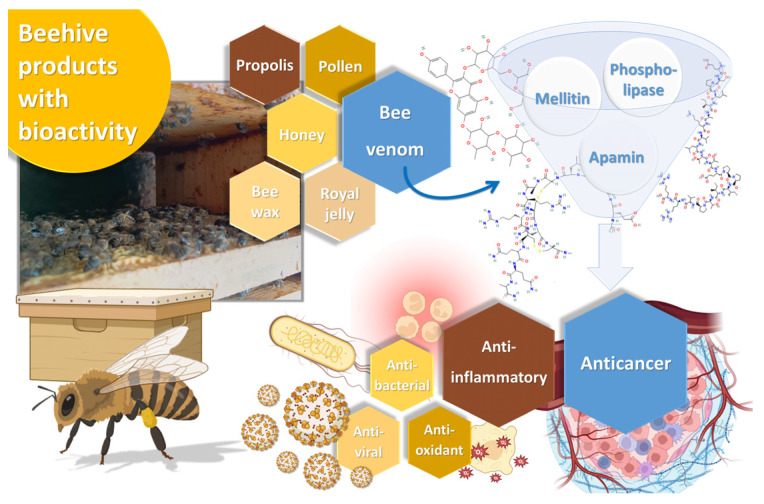
Schematic representation of beehive derived compounds, including a photograph of the beehive at Polytechnic Institute of Bragança’s apiary, Bragança, Portugal (produced with Biorender, melittin, phospholipase and apamin molecular structures originated from PubChem).

In this context, natural-derived products might be the answer [18,19,20]. Bee venom, also known as apitoxin, is a substance produced in the venom gland under the abdominal cavity of female worker bees, and it is a colorless and odorless liquid, with an acid pH between 4.5 and 5.5. It is used by these insects to defend the hive against external threats [21]. In its composition, we can find peptides like melittin, apamin, adolapin, and scapin, and enzymes such as phospholipase A2, hyaluronidase, and lysophospholipase, as well as other substances, including amino acids, carbohydrates, pheromones, and minerals. Among all these compounds, melittin is the main active ingredient in bee venom, comprising 40% to 60% of its dry weight, followed by the enzyme phospholipase A2, present in 10–12% [22]. The complex chemical composition of bee venom, and the potential synergy surrounding the interaction between its compounds, assures a vast range of biological activities, capable of targeting different diseases. These biological properties include anti-inflammatory, antioxidant, antiviral, antimicrobial and antitumor properties (Figure 1) [23,24]. All these bioactivities could be potentially beneficial for a series of different diseases, from skin diseases, such as acne, wounds, psoriasis, or atopic dermatitis, to neurodegenerative diseases, such as Parkinson’s disease, due to proven neuroprotective effects, and, of course, cancer [25,26]. Nevertheless, while bee venom can be tolerated by the human body, it can cause some allergic reactions, as well as systemic and local inflammatory responses, other immune reactions, and anticoagulant effects, which are the main drawbacks for its use as a potential therapeutic agent [22]. Bee venom toxicity has been reported to be concentration-dependent on blood cells, namely in human lymphocytes, leading to the induction of oxidative stress-related DNA damage, and, consequently, cellular instability, and overall genotoxicity, making this one of the most relevant barriers to its use for therapeutic purposes [27,28,29]. In this context, new strategies are needed in order to increase the safety of bee venom administration and its incorporation into nanotechnological platforms to enhance its properties, avoid degradation, and reduce the potential side effects [30].

The use of nanotechnology for drug delivery can not only protect the drug from chemical and metabolic degradation, but also allow increased permeation through biological barriers, and enable a controlled, sustained, and targeted drug delivery, hence leading to more localized therapeutic effects, and thus reduced systemic toxicity [31,32,33]. Several types of nanosystems have been developed over the years, namely nanoemulsions, micelles, polymeric nanoparticles, and lipid nanoparticles, such as liposomes, and their more recent and innovative alternatives, ethosomes, transfersomes and niosomes [34,35,36]. Niosomes are vesicular nanosystems capable of self-assembly, composed of amphiphilic molecules, such as non-ionic surfactants, which form an outer bilayered membrane, and an aqueous core [37,38]. This structure makes them highly versatile nanosystems for therapeutic application since they can deliver both hydrophobic molecules, within the bilayered membrane, and hydrophilic molecules, encapsulated within their aqueous core. Other molecules, such as cholesterol or similar lipids, can also be part of the niosomal membrane, giving them better properties, such as higher stability and deformability [39,40]. Thanks to their composition, niosomes are biodegradable and biocompatible, on account of the similarity between the lipids used for niosome production and the ones found in the human body, especially on the cellular membranes [41,42]. Additionally, when compared to their ancestors, liposomes, niosomes possess higher stability and longer shelf-life, a better capacity for controlled drug release, and lead to higher drug bioavailability [43].

Hence, given the great therapeutic potential of bee venom, and the promising properties of niosomes for increased therapeutic efficacy and safety, the purpose of the present study was to develop and characterize bee venom-loaded niosomes, for potential anticancer therapy. Their physicochemical, therapeutic and safety profiles were assessed, including particle size and polydispersity index determination, in vitro cytotoxicity, and anti-inflammatory potential assessment, and in vitro and ex vivo safety evaluation. Limitations regarding these assays (including the assessment of the optimum hydration temperature for niosome production, determination of the ideal dilution for particle size measurement, and the determination of the adequate positive and negative controls for the cell assays) were thoroughly assessed and resolved.

## 2. Results and Discussion

### 2.1. Niosomal Formulation Development and Characterization

**Figure 2:**
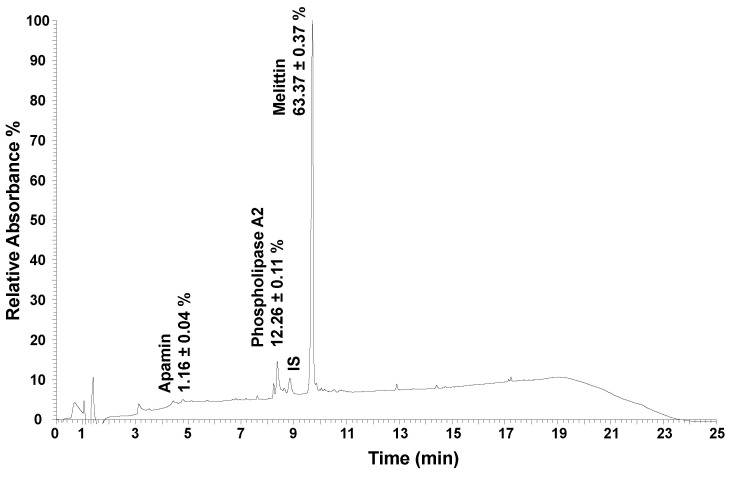
Example chromatogram after UHPLC analysis of the bee venom sample, at 220 nm. IS—internal standard (cytochrome C, 25 µg/mL); UHPLC—ultra-high-performance liquid chromatography.

The bee venom samples went through chemical characterization, by quantification of its major constituents (Table 1) [44]. An example of the obtained chromatogram using UHPLC is shown in Figure 2. Three main compounds were quantified, with melittin being the most abundant compound present in the sample (63%), followed by phospholipase A2 (12%), and apamin (1.2%), which is in agreement with the reported previous works [21,45].

Tween^®^ 20, a hydrophilic non-ionic surfactant, was used to form the niosomes, with the addition of cholesterol and cetyl alcohol (1:1) as the lipidic components. It has been reported that non-ionic surfactants with HLB values above 6 require the addition of a lipidic component to correctly produce niosomes [38]. Additionally, adding cholesterol to a niosomal formulation improves the cohesion of the niosomal membrane [46], enhances membrane stability, decreases membrane fluidity, eliminates the gel-to-liquid phase transition temperature, allows better loading of hydrophilic drugs [47], and improves entrapment efficiency by filling empty spaces in the bilayer vesicle [42]. The addition of cholesterol and cetyl alcohol has also been reported to improve the overall stability of the produced niosomal structures [48].

**Figure 3:**
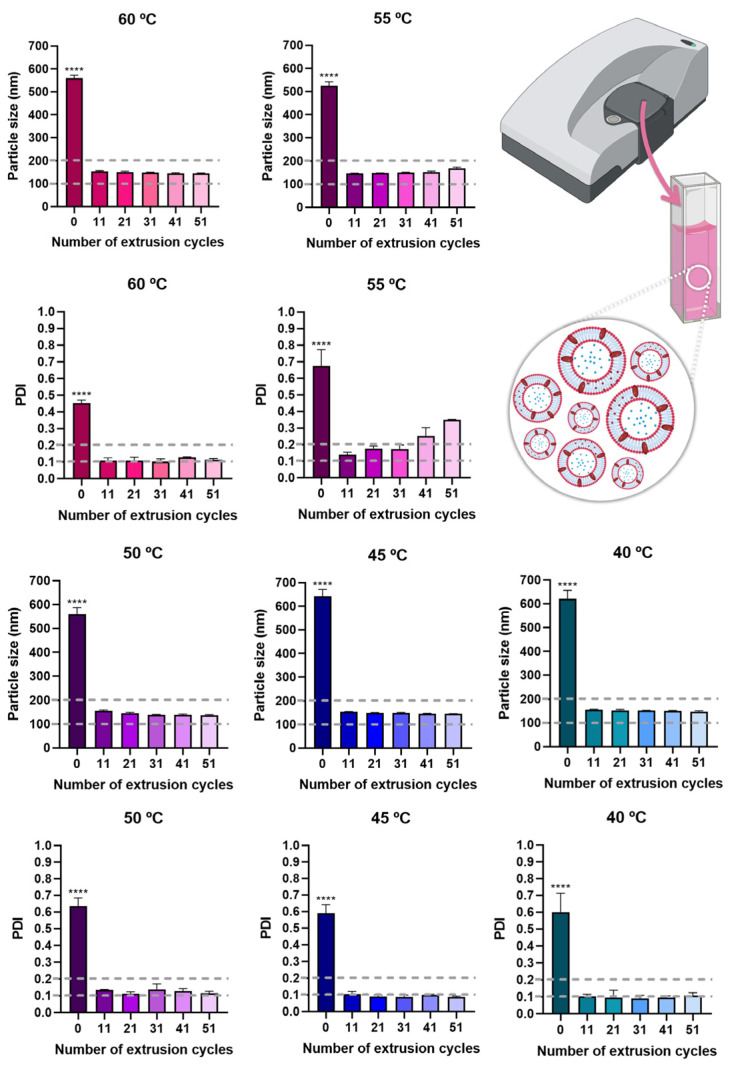
Particle size and PDI values of the developed niosomes (formulation vehicle), at all tested temperatures, and with a varying number of performed extrusion cycles; data is presented as mean ± standard deviation; **** *p* < 0.0001 and corresponds to the comparison of no extrusion with all extrusion cycles (R^2^ 0.9991, one-way ANOVA with Tukey’s multiple comparisons test); PDI—polydispersity index.

As the main composition of bee venom is proteins, these can denature at high temperatures, hence during production, an effort should be made to use the lowest temperatures possible [49]. For this reason, multiple hydration temperatures were tested, namely 40 °C, 45 °C, 50 °C, 55 °C, and 60 °C. The results showed that after extrusion, similar particle size and PDI values were obtained for all hydration temperatures (Figure 3), and hence the lowest temperature, 40 °C, was selected. Additionally, although there was a statistically significant difference in particle size and PDI before extrusion and after extrusion (*p* < 0.0001, R^2^ 0.9991, one-way ANOVA with Tukey’s multiple comparisons test), all extrusion cycles originated a particle size below 200 nm, which is suitable for all kinds of administration routes, making the formulation highly versatile in what concerns potential application. PDI values were also always below 0.3, for all extrusion cycles and temperatures, and in most cases also below or equal to 0.2, making the formulations highly homogeneous [50,51,52]. Therefore, for the selected temperature, 40 °C, the number of extrusion cycles which originated the lowest particle size and PDI values was selected, being equal to 31 extrusion cycles. These niosomes were hence selected for compound encapsulation and had a particle size of 151.2 ± 1.8 nm and a PDI of 0.091 ± 0.018 (Table 2). As for the zeta potential of the optimized formulation, it was between −30 and −40 mV, which could have a contribution in formulation stabilization through electrostatic repulsion.

Once the niosomes were optimized (formulation vehicle, still no encapsulated compound), bee venom was added to the vesicles’ aqueous core, to be encapsulated at a concentration of 2 µg/mL. This concentration was selected based on previous studies, for it to be enough to have bioactivity, but not so much that it would be potentially toxic. According to previous studies, when using concentrations of bee venom of 3.125 µg/mL or 5 µg/mL, a substantial decrease in healthy cells’ viability was observed, with values ranging from only around 50 to 60% [53]. Hence, a lower value was selected, to help guarantee formulation safety.

After the encapsulation of bee venom, the mean particle size was equal to 150.4 ± 3.7 nm, remaining the same as without compound encapsulation, and the PDI was equal to 0.162 ± 0.008, hence having a slight increase (as had been reported by previous studies [54]), but still being below 0.2, and therefore still constituting a quite homogeneous nanosystem (Table 2).

### 2.2. In Vitro Cytotoxic Potential

**Figure 4:**
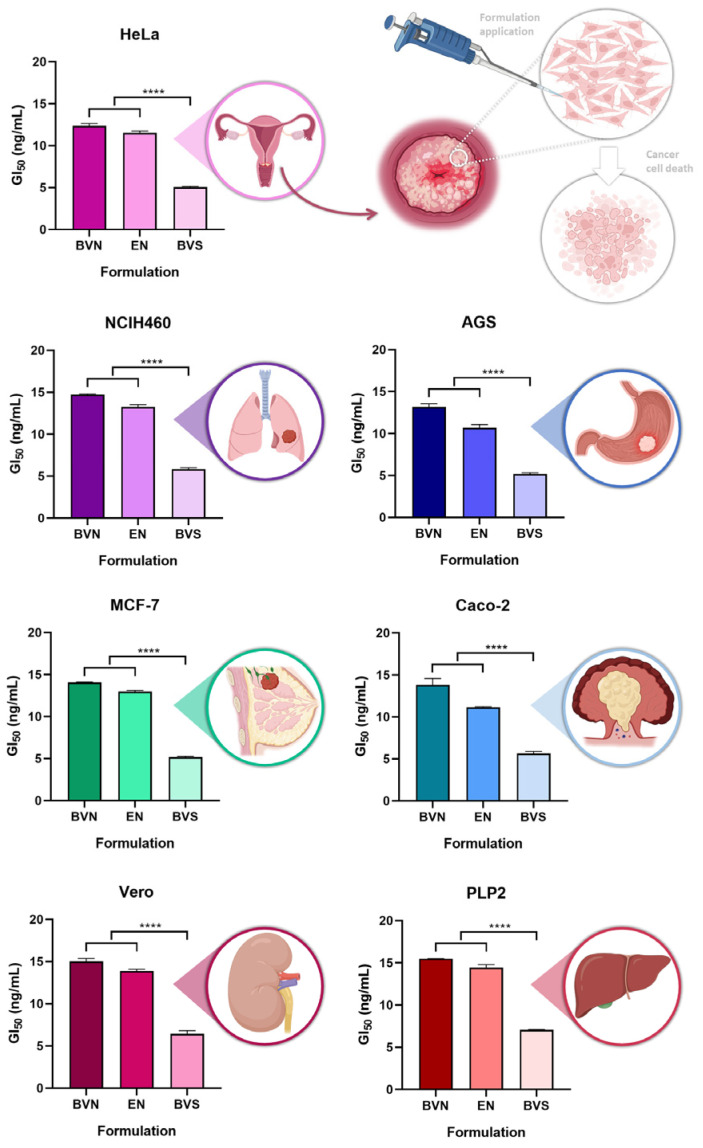
Cytotoxic potential of the developed bee venom-loaded niosomes, compared to the empty niosomes (formulation vehicle) and bee venom solution, in several different cancer cell lines; GI_50_ corresponds to the formulation concentration required to inhibit cell growth by 50%; data is represented as mean ± standard deviation; **** *p* < 0.0001 (R^2^ 0.9991, one-way ANOVA with Tukey’s multiple comparisons test); AGS—gastric adenocarcinoma cell line; Caco-2—colorectal adenocarcinoma cell line; HeLa—cervical carcinoma cell line; MCF-7—breast adenocarcinoma cell line; NCI-H460—lung carcinoma cell line; PLP2—primary pig liver culture cell line; and Vero—African green monkey kidney cell line.

The in vitro cytotoxic potential of the developed bee venom-loaded niosomes was tested, at different concentrations, in different cell lines, with the cytotoxicity being evaluated using a sulforhodamine B colorimetric assay, in five human tumor cell lines, namely AGS (gastric adenocarcinoma), Caco-2 (colorectal adenocarcinoma), MCF-7 (breast adenocarcinoma), NCI-H460 (lung carcinoma) and HeLa (cervical carcinoma). Two non-tumor cell lines were also used, Vero (African green monkey kidney) and PLP2 (primary pig liver culture), to assess for potential systemic toxicity. Results are shown in Figure 4.

A substantial anticancer potential has been previously reported for bee venom, related to its capacity to inhibit cancer cell growth, and induce apoptosis, and more specifically related to its major components melittin and phospholipase A2 [55,56]. Other studies have demonstrated that bee venom can trigger regional cellular immune reactions in lymph nodes, exerting its effects through not only apoptosis, but also lysis mechanisms and tumor cell necrosis [57,58]. Our results show that the bee venom solution had the most significant cytotoxic effect, both against the studied cancer cell lines, and the healthy cell lines, attributed to its lower GI_50_ values, ranging from 5.05 to 5.87 ng/mL (*p* < 0.0001, R^2^ 0.9991, one-way ANOVA with Tukey’s multiple comparisons test). Although this means that the bee venom solution has a higher anticancer potential while unencapsulated, it also means that it has a higher potential for toxicity in healthy tissues, hence being connected to a higher propensity for systemic side effects. Hence, compound encapsulation will reduce its potentially harmful effects in healthy cells, while still having substantial anticancer potential in tumor cell lines. Hence, when encapsulated into the niosomes, a higher targeting potential to cancer cells can be obtained, while being able to avoid destroying healthy cells.

Regarding the AGS cell line, the bee venom-loaded niosomes present a GI_50_ value of 13.19 ng/mL, still demonstrating a good cytotoxic potential after bee venom encapsulation. A previous study [59] has shown that melittin present in bee venom can stop AGS cell proliferation even at low concentrations. Concerning the Caco-2 cell line, the bee venom-loaded niosomes present a GI_50_ value of 13.82 ng/mL, thereby retaining the cytotoxic potential of bee venom after encapsulation. This anticancer activity against the Caco-2 cell line was also demonstrated in a previous study [60], where the developed nanoparticles were able to deliver the active compound to the correct target, the cancer cells. In what concerns the MCF-7 cell line, the bee venom-loaded niosomes presented a GI_50_ value of 14.05 ng/mL, thereby being substantially cytotoxic for this cell line as well. Bee venom has been proven to inhibit the growth of breast cancer cells even in small concentrations, as observed in previous studies [61]. For the NCIH460 cell line, the bee venom-loaded niosomes showed a GI_50_ value of 14.72 ng/mL, hence demonstrating good anticancer potential for this cell line too. Bee venom has also been proven to inhibit cell proliferation of NCIH460 cells in a concentration-dependent manner in prior studies [62]. Regarding the HeLa cell line, the bee venom-loaded niosomes presented a GI_50_ value of 12.37 ng/mL, still demonstrating a considerable cytotoxic potential even after compound encapsulation. Bee venom has also demonstrated anticancer properties in these cells previously, in a concentration-dependent manner [63].

As for previous similar studies, regarding nanosystem development for therapeutic purposes containing bee venom, a prior study where a nanoliposome was designed to encapsulate bee venom with similar concentrations was tested in some cancer cell lines, in which a cytotoxicity assay was also performed [64]. In comparison, for our study, smaller concentrations of bee venom were necessary to inhibit the growth of cancer cells by 50%. Hence, overall, the obtained results showed that the developed bee venom-loaded niosomes could serve as a potential adjuvant treatment for several types of cancer, including cervical, breast, lung, stomach, and colorectal carcinomas. Bee venom can be used as a therapeutic agent for cancer thanks to its complex chemical composition, and even when encapsulated into niosomes, bee venom has presented good cytotoxic properties against various cancer cells, in this case enhancing its anticancer potential by providing a controlled and sustained release of the compound, ideal for a prolonged therapeutic effect.

### 2.3. In Vitro Anti-Inflammatory Activity

In recent years, the link between inflammation and cancer has been extensively studied, both in what concerns pathogenesis and therapeutics [65,66,67]. Inflammation has been proven to increase the chances of cancer development, promoting all stages of tumorigenesis, and not only regulating cancer development but also therapeutic responses, with chronic inflammation having an important role in both stimulating tumor progression and leading to treatment resistance [68,69,70]. Cancer cells and inflammatory cells have been proven to interact to form an inflammatory tumor microenvironment, with inflammatory factors and metabolites such as several cytokines (tumor necrosis factor (TNF)), interleukins (IL), interferons (IFN)), chemokines, growth factors, inflammasomes, leukotrienes, prostaglandins, and thromboxane being identified as having important roles in the initiation and regulation of cancer-related inflammatory processes [68,69,71]. Additionally, inflammation has been linked to DNA damage in cancer stem-like cells, leading not only to the development of cancer, but to more aggressive forms of the disease, and also being connected to increased oxidative stress, with the generation of reactive oxygen and nitrogen species which will also damage and lead to dysfunctional lipids and proteins [72,73,74]. For these reasons, compounds with anti-inflammatory action have been studied as alternative or adjuvant treatments for all types of cancers [75,76,77].

**Figure 5:**
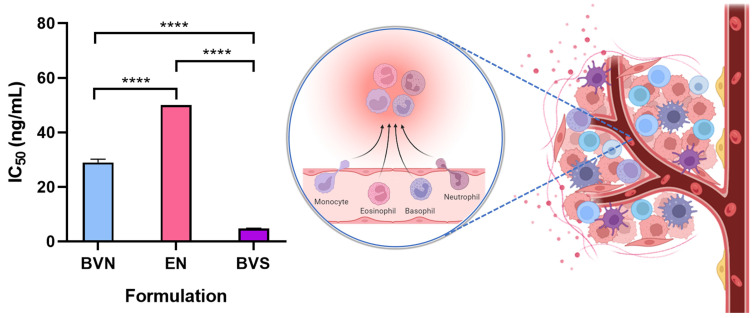
Anti-inflammatory potential of the developed bee venom-loaded niosomes (BVN), compared to the free compound (bee venom solution, BVS), and the formulation vehicle (empty niosomes, EN), evaluated in a mouse macrophage cell line (RAW 264.7); IC_50_ values are depicted, and correspond to formulation concentrations providing 50% of inhibition of nitric oxide production; data is represented as mean ± standard deviation; **** *p* < 0.0001 (schematic representation produced with Biorender).

Hence, to assess the anti-inflammatory properties of the developed bee venom-loaded niosomes, for potential synergistic effects with the non-inflammatory related anticancer potential of the formulation, the pro-inflammatory response was evaluated in the mouse macrophage cell line RAW 264.7 (Figure 5). The developed bee venom-loaded formulation was compared with the free compound (bee venom solution), and the formulation vehicle. The IC_50_ (concentration of the formulation required to inhibit 50% of nitric oxide production) was determined and calculated, as a demonstration of its anti-inflammatory activity.

The bee venom solution demonstrated an IC_50_ of 4.77 ng/mL, the lowest out of the compared formulations (*p* < 0.0001, R^2^ 0.9991, one-way ANOVA with Tukey’s multiple comparisons test), which is indicative of an extensive anti-inflammatory potential, and in line with previous results from the research team [78]. Additionally, this potential has been further confirmed by other past publications, where similar bee venom concentrations depicted similar anti-inflammatory properties, with its main constituent, melittin, being responsible for the majority of such effects [79,80,81]. In what concerns specific action mechanisms, in previous studies, bee venom proved to have significant anti-inflammatory effects not only by inhibiting phospholipase A, but also by decreasing pro-inflammatory cytokines IL-1β, IL-6, and IFN-γ, and chemokines CCL22 and CCL17 levels, in in vitro assays [82,83,84]. Moreover, in an in vivo model of atopic dermatitis, bee venom proved to significantly reduce IL-1β, IL-4, IL-6, IL-13, immunoglobulin E (IgE) and TNF-α levels, also leading to a reduction in the number of inflammatory cells (neutrophils, eosinophils, monocytes), and cyclooxygenase-2 (COX-2) inhibition [84,85,86]. Also in vivo, in an arthritis animal model, bee venom has proven to have anti-inflammatory effects by inhibiting IL-1β, IL-6, TNF-α and TGF-β1 levels, as well as having complementary antioxidant effects, with a reduction in total antioxidant status, and leading to a reduction in DNA damage [21,87]. Further supporting these in vitro and in vivo results, several clinical trials with bee venom administration to inflammatory-based diseases’ patients, such as rheumatoid arthritis, adhesive capsulitis, and pelvic inflammatory disease, have demonstrated promising outcomes, with substantial improvement of clinical symptoms and disease markers, after topical or intravenous administration [88,89,90,91]. Although specific molecular mechanisms are still in need of deeper exploration, these results have been suggested to be connected to COX-2 and prostaglandin E2 inhibition, among other mechanisms [88,92].

Furthermore, as expected, the formulation vehicle (empty niosomes) showed no substantial anti-inflammatory potential, with an IC_50_ value of >50 ng/mL, the highest out of the studied formulations (*p* < 0.0001, R^2^ 0.9991, one-way ANOVA with Tukey’s multiple comparisons test), confirming that none of the components used in niosome composition depict intrinsic anti-inflammatory effects. As for the bee venom-loaded niosomes, the formulation presented an IC_50_ of 28.98 ng/mL, which albeit is higher than that obtained for the bee venom solution (*p* < 0.0001, R^2^ 0.9991, one-way ANOVA with Tukey’s multiple comparisons test), can still be considered a relevant anti-inflammatory effect, meaning that when encapsulated into the nanometric structures the bee venom does not lose its anti-inflammatory potential. Additionally, the higher IC_50_ value of the bee venom-loaded niosomes when compared to the free compound is likely related to the controlled release of the molecule from within the nanosystems, potentially leading to a sustained and prolonged therapeutic effect, which has been shown to produce enhanced bioavailability in previous studies, using similar delivery systems and compounds [93,94,95].

### 2.4. In Vitro Safety Assessment—Cytotoxicity Evaluation on HaCaT and HFF-1 Cell Lines

**Figure 6:**
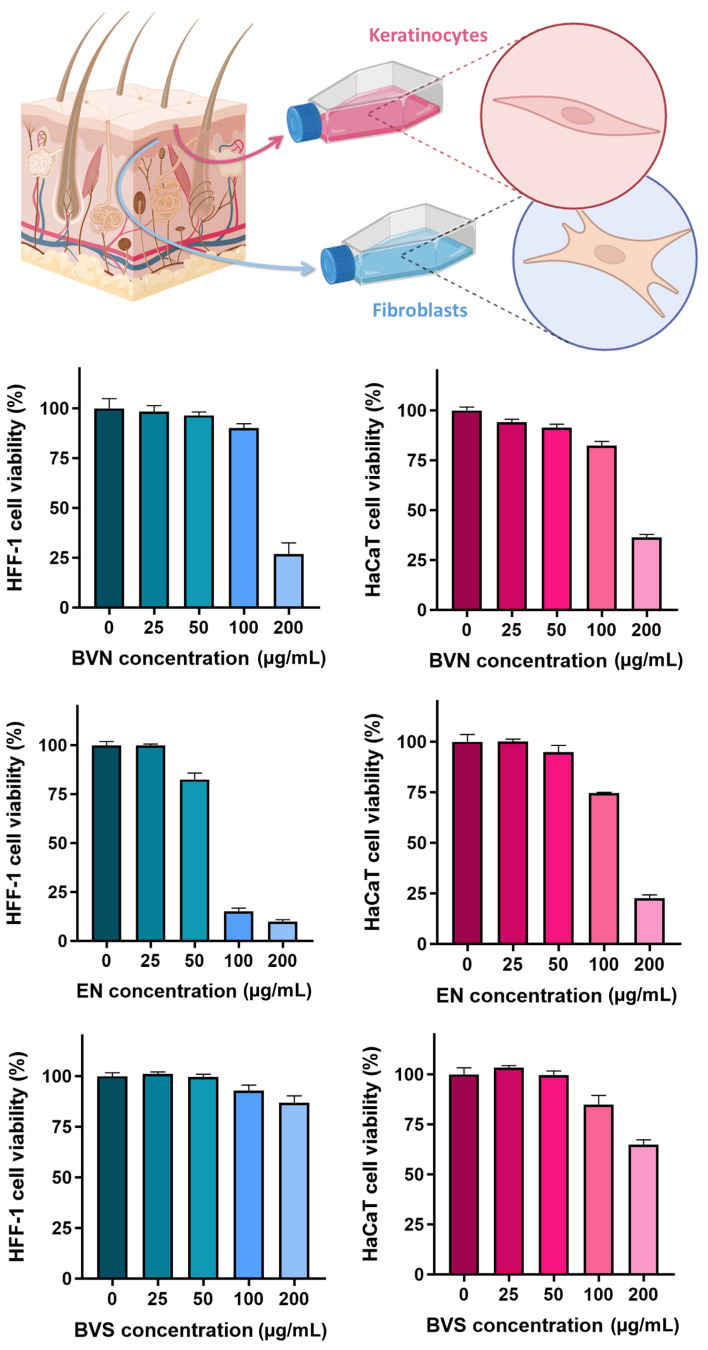
Cell viability percentage (%) variation with increasing applied formulation concentrations (µg/mL), on HFF-1 cell line (left bar graphs, in blue), and HaCaT cell line (right bar graphs, in pink), including the developed bee venom loaded niosomes (BVN), formulation vehicle (empty niosomes, EN), and compound solution (bee venom solution, BVS); data is represented as mean ± standard deviation; HaCaT—skin keratinocytes cell line; HFF-1—skin fibroblasts cell line.

Assays evaluating formulation safety in skin cellular models have long been applied to predict potential toxicity issues, being especially relevant for intended transdermal or topical administration [96,97,98]. Hence, to evaluate the safety of the developed bee venom-loaded niosomes and compare them to the vehicle formulation (empty niosomes), and free compound (bee venom solution), a sulforhodamine B colorimetric assay was conducted, in two human skin cell lines, HaCaT (keratinocytes) and HFF-1 (fibroblasts). The results, depicted in Figure 6, showed significant differences between concentrations within the same formulation (statistical significance matrix on Table 3), and also when comparing the same concentration for different formulations (Table 4).

Overall, the results show that there is a concentration-dependent safety of the developed formulations (*p* < 0.0001, two-way ANOVA with Tukey’s multiple comparisons’ test), with the safest concentration in both HaCaT and HFF-1 cell lines appearing to reside between 50 and 100 µg/mL. Specifically for the bee venom solution, safety appears to be related to concentrations up to 200 µg/mL in the HFF-1 cell line, but only up to 100 µg/mL in the HaCaT cell line. These results are in accordance with the ones obtained by previous studies for the HaCaT cell line [99], where cell viability tended to reduce with increasing bee venom concentrations, and with safety existing up to 100 µg/mL, and appear to be more promising than the ones reported by previous studies for the HFF-1 cell line [100], where only concentrations equal or lower than 10 µg/mL were considered safe. Additionally, IC_50_ values (formulation concentrations causing 50% reduction of cell viability or proliferation, Table 5) were proven to be 165.7 ± 1.0 µg/mL and 161.0 ± 1.0 µg/mL for the bee venom-loaded niosomes, 138.2 ± 1.0 µg/mL and 69.8 ± 1.0 µg/mL for the formulation vehicle, and >200.0 µg/mL for the bee venom solution, in HaCaT and HFF-1 cells, respectively.

Furthermore, the results showed that the developed niosomes without bee venom (formulation vehicle) appear to be less safe than the bee venom-loaded niosomes, in both cell lines (*p* < 0.0001, two-way ANOVA with Tukey’s multiple comparisons’ test). This seemingly unexpected cytotoxicity of the empty niosomes might be related to the quantities of the excipients used to formulate them. A previous study, on polysorbate 20 (Tween 20) formulations applied on HaCaT cells, showed that at elevated concentrations cytotoxicity could be detected, as well as after long periods of exposure [101]. Additionally, the lesser cytotoxicity of bee venom-loaded niosomes could be due to the absorption of the hydrophilic compound to the surface of the vesicles, thereby decreasing their toxicity due to decreasing the direct contact of the formulation compounds with the cells.

Hence, overall, the obtained results showed that the developed niosomes can only be administered on the skin up to certain concentrations, but since in vitro results have been reported to not always be directly related to what happens in vivo [102,103], and, even more, in a clinical context [104,105], further studies would have to be performed to assess the true safety of the developed nanocarriers. Therefore, safety was further assessed in the HET-CAM test (results shown in the following Section 2.5). It is also relevant to mention that given the known severe side effects related to the grand majority of anti-cancer therapies [106,107,108], a risk-benefit ratio would always have to be assessed if the developed formulations are proven to be of great therapeutic potential as primary or adjuvant anticancer therapies.

### 2.5. Ex Vivo Safety Assessment—HET-CAM Test

**Figure 7:**
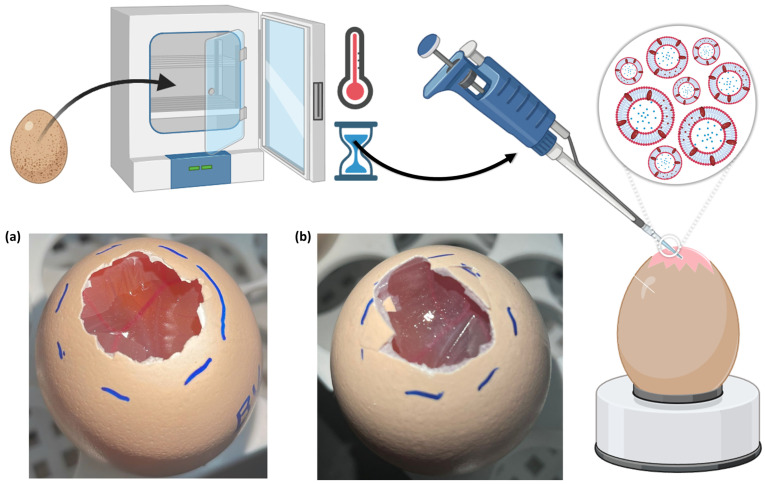
HET-CAM assay schematic representation, and photographs of the hen’s egg chorioallantoic membranes after formulation application, either the developed bee venom-encapsulated niosomes (**a**), or the formulation vehicle (empty niosomes) (**b**); HET-CAM—hen’s egg chorioallantoic membrane.

The HET-CAM test has been used for many years as a useful model to predict the potential of developed formulations to cause eye irritation, hence making the preparations that are proven to be safe after application onto the hen’s egg chorioallantoic membranes, also potentially safe for administration through most administration routes, given that the eye is one of the most sensitive organs in the human body [109,110,111]. Hence, to evaluate the irritation degree of the developed bee venom-loaded niosomes, a HET-CAM test was conducted, with the application of 0.3 mL of each formulation (non-diluted) onto each fertilized egg’s chorioallantoic membrane. Then, during the first 5 min after application, the membrane was observed for vascular reactions in the blood vessels, according to the described method’s protocol [112]. Results are shown in Table 6 and Figure 7.

Results showed that, as expected, no irritability was observed when 0.9% NaCl (negative control) was applied, and strong irritability was observed when 0.1 N NaOH (positive control) was applied. Furthermore, and in accordance with the results obtained from the in vitro cytotoxicity assays (Section 2.2), the empty niosomes showed a strongly irritative degree, with an IS value of 14, and caused hemorrhage 5 min after formulation application (Figure 7b). Again, just like in the in vitro assays, the unexpectedly high irritation degree of the empty niosomes could be related to its constituents, as some data has been found regarding the irritability of Tween 20 and cholesterol [113,114]. Additionally, in a previous study [115], where two different Tween 20 concentrations were tested (30% and 1%), results showed that higher concentrations of this non-ionic surfactant caused a higher irritation score than lower concentrations. Nevertheless, the bee venom-loaded niosomes had a much smaller irritative potential, with an IS value of 7.7, being half of the obtained for the empty niosomes, which indicates only a slight irritative degree, with no associated lysis or hemorrhage. Previous studies [116] have reported that bee venom, and more specifically melittin, can cause irritation in a dose-dependent manner. Hence, it seems that the bee venom concentrations used in the developed nanocarrier’s composition appear to be safe. Moreover, and once again, the much lower IS obtained with the bee venom-loaded niosomes, when compared to the empty niosomes, could be due to the adsorption of some of the compound onto the vesicles’ outer membrane, making the direct contact between the chorioallantoic membrane and the niosomes more reduced, and hence lowering the potential for irritation.

Therefore, since the bee venom-loaded niosomes showed hen’s egg chorioallantoic membranes with normal blood vessels, with no lysis or other vascular reactions, and an overall low IS, the developed nanocarrier appears to have a reasonable safety profile. Nevertheless, again, additional tests are needed to confirm these results, to find the safest and, yet, most therapeutically effective concentrations of the developed formulation, with the assessment of a risk-benefit ratio being necessary to confirm the true value of the developed nanocarriers as primary or adjuvant anticancer therapies.

## 3. Materials and Methods

### 3.1. Materials and Reagents

Tween^®^ 20, cholesterol, cetyl alcohol, chloroform, sulforhodamine B, lipopolysaccharide, trypan blue, dexamethasone, trichloroacetic acid, tris (hydroxymethyl)aminomethane buffer, cytochrome C from equine heart (purity ≥ 95%), melittin (purity ≥ 85%, HPLC grade) and phospholipase A2 (activity: 1775 units/mg solid) were purchased from Sigma-Aldrich (Saint Louis, MO, USA). Apamin (purity 98.3%) was purchased from CalBiochem (San Diego, CA, USA). Fetal bovine serum, penicillin, streptomycin, trypsin, L-glutamine, and Dulbecco’s modified Eagle’s medium (DMEM) were purchased from Gibco Invitrogen Life Technologies (Carlsbad, CA, USA). Formic acid (HPLC grade) and acetonitrile (HPLC grade) were obtained from Fisher Scientific (Loughborough, UK). The Griess reagent system kit was bought from Promega (Madison, WI, USA). Ultrapure water was obtained from adequate purification systems (Ellix Essential Millipore^®^, Darmstadt, Germany, and TGI Pure Water Systems, Brea, CA, USA).

### 3.2. Cell Lines

All human and animal cell lines used in this work are commercially available and were purchased from different authorized cell line resources, including the German Collection of Microorganisms and Cell Cultures (DSMZ) and the European Collection of Authenticated Cell Cultures (ECCAC). The human tumor cell lines used for the cytotoxicity assays included: MCF-7 (breast cancer), NCI-H460 (non-small-cell lung cancer), AGS (gastric adenocarcinoma), HeLa (cervical cancer) and Caco-2 (colorectal adenocarcinoma). All these cell lines were obtained from DSMZ, except the Caco-2 cell line, which was obtained from the ECACC. The non-tumoral macrophage derived cell line RAW 264.7, used for the anti-inflammatory in vitro studies, was purchased from the ECACC. Primary cell lines, obtained from porcine liver tissue (PLP2) and African green monkey kidney (Vero), were also used to study the cytotoxicity effect in non-tumoral cells. In order to maintain high scientific standards, all procedures were performed according to the best practices observed in the Guidance on Good Cell Culture Practice (GCCP).

### 3.3. Bee Venom Production and Harvesting

The bee venom used as the active compound to be encapsulated within the developed niosomes was collected between August and November of 2018 from *Apis mellifera intermissa* hives located in the northeast region of Morocco. To collect the bee venom, a double-face bee venom collector was used, especially developed for the purpose by the research team. The device was positioned in the hive at one of the outermost, diametrically opposed ends of the beehive, and produced mild electrical impulse shock waves on the beehive, which made the worker bees sting the glass, as a defense mechanism, leaving the bee venom deposited on it. Following the collection process, the venom was meticulously removed from the glass using a scraper and subsequently stored in pharmaceutical-grade vials. Samples were then freeze-dried, in a freeze dryer (Labconco FreeZone 4.5, Labconco Corporation, Kansas City, MO, USA), and kept at −20 °C until further analysis [78].

### 3.4. Ultra-High-Performance Liquid Chromatography Analysis

The ultra-high-performance liquid chromatography (UHPLC) analysis was executed utilizing a Dionex Ultimate 3000 UHPLC instrument (Thermo Scientific, Waltham, MA, USA), featuring a diode-array detector. The chromatographic system comprised a quaternary pump, an autosampler maintained at 5 °C, a degasser, a photodiode array detector, and an automatic thermostatic column compartment. Chromatographic separation was conducted on an XSelect CSH130 C18, 100 mm × 2.1 mm id, 2.5 µm XP column (Waters, Milford, MA, USA), with a constant temperature of 30 °C. The mobile phase consisted of 0.1% (*v*/*v*) formic acid in water and 0.1% (*v*/*v*) formic acid in acetonitrile, previously degassed and filtered. The used conditions were in accordance with previous studies [44]. Spectral data for all peaks were gathered within the range of 190–500 nm. Control and data acquisition were conducted using the Xcalibur^®^ data system (Thermo Scientific, Waltham, MA, USA). Cytochrome C, employed as the internal standard, was prepared in deionized water at a concentration of 25 µg/mL. For analysis, the lyophilized bee venom (3 mg) was dissolved in 10 mL of internal standard. Each sample was filtered through a 0.2 µm polytetrafluoroethylene membrane. Bee venom peptide quantification was achieved using calibration curves for apamin (at a range of 2–60 µg/mL, y = 0.040x + 0.055, R^2^ = 0.999), phospholipase A2 (at a range of 8–120 µg/mL, y = 0.049x − 0.356, R^2^ = 0.999), and melittin (at a range of 15–250 µg/mL, y = 0.062x + 0.029, R^2^ = 0.997).

### 3.5. Niosome Formulation Development and Characterization

**Figure 8:**
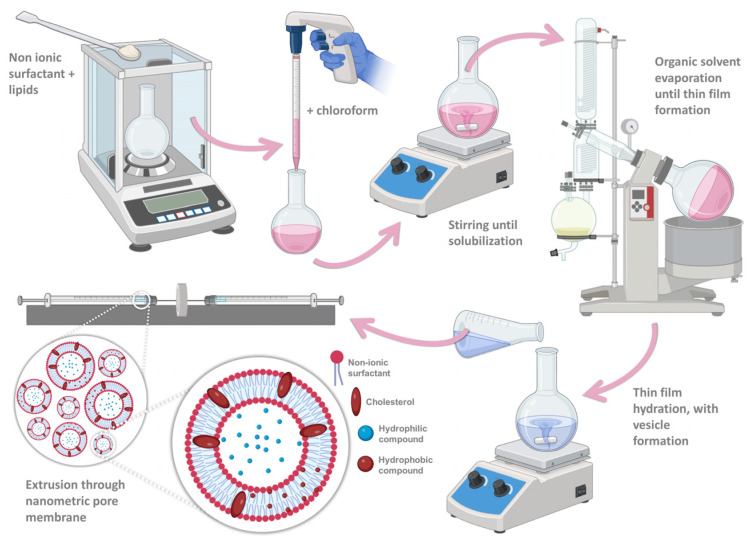
Schematic illustration of niosome composition and production, using the thin-film hydration method, followed by extrusion through a nanometric pore membrane (produced with Biorender).

For formulation preparation, the thin-film hydration method was applied (Figure 8), which is the most commonly used method for niosome production [38,117]. Firstly, the non-ionic surfactant and lipid fraction (3.5:2 molar ratio) were weighed into a round bottom flask and then dissolved in 6 mL of chloroform. The organic solvent was subsequently evaporated, in a rotary evaporator (Rotavapor R-210/215, BÜCHI, Meierseggstrasse, Flawil, Switzerland), combined with a vacuum pump (V-700/710, BÜCHI, Switzerland), and a vacuum controller (V-850/855, BÜCHI, Switzerland), in a heating water bath (40 °C to 60 °C), with a rotation speed of 8 rpm, and under reduced pressure (120 mbar), for 60 min. After full organic solvent evaporation, a thin layer was formed on the interior of the flask, and this thin layer was then hydrated with either deionized water (6 mL) for the formulation vehicle (empty niosomes), or an aqueous compound solution (2 µg/mL) for the bee venom-loaded niosomes, under mild magnetic stirring, for 60 min. Although after thin-film hydration vesicles were already formed, to attain a nanometric and homogeneous particle size, the mixture was extruded (Avanti Mini-Extruder, Avanti Polar Lipids, Alabaster, AL, USA) through a synthetic polycarbonate membrane with a 200 nm pore size (Avanti Polar Lipids, USA). Various extrusion cycles were performed (11, 21, 31, 41, and 51), and the particle size and polydispersity index (PDI) for every performed cycle series were subsequently measured. Particle mean hydrodynamic size and PDI of the developed formulations were measured by dynamic light scattering, using a Zetasizer apparatus (ZetaSizer Nano ZS, Malvern Instruments, Malvern, UK). Samples were diluted 40-fold in deionized water and measured in disposable polymethyl methacrylate 12 mm square diameter cuvettes, at 25 °C. Zeta potential was also measured using the same apparatus, using a folded capillary cell (DTS1070), through electrophoretic light scattering. All the samples were measured in triplicate and the mean and standard deviation are reported. Formulations were stored in a refrigerator at 4 °C until further characterization.

### 3.6. In Vitro Therapeutic Potential

#### 3.6.1. Cytotoxic Activity

The developed formulations’ cytotoxic activity was tested in several different human cancer cell lines, namely: AGS (gastric adenocarcinoma), Caco-2 (colorectal adenocarcinoma), MCF-7 (breast adenocarcinoma), NCI-H460 (lung carcinoma), and HeLa (cervical carcinoma). For assessment of the potential toxicity of the developed formulations on non-cancerous tissues, non-tumor cell lines Vero (African green monkey kidney) and PLP2 (primary pig liver culture) were also tested. All cell lines were maintained in RPMI-1640 medium, supplemented with 10% fetal bovine serum, glutamine (2 mM), penicillin (100 U/mL), and streptomycin (100 mg/mL), except Vero cells, which were maintained in DMEM medium. The culture flasks were incubated at 37 °C, under a 5% CO_2_ and high humidity atmosphere. Cells were used only upon reaching a 70 to 80% confluence. 

Formulations were dissolved in water: dimethyl sulfoxide (DMSO) (1 mL; 1:1) to obtain stock solutions with a concentration of 1 μg/mL, from which successive dilutions were made, obtaining the samples at the concentrations to be tested (0.05–0.0008 μg/mL). Each sample (10 μL) was incubated with the cell suspension (190 μL) in 96-well microplates, for 72 h. The microplates were incubated at 37 °C, under a 5% CO_2_ and high humidity atmosphere. All cell lines were tested at a concentration of 10,000 cells/well, except for Vero cells, in which a density of 19,000 cells/well was used. Subsequently to this incubation period, plates were incubated again, for 1 h, at 4 °C, after the addition of previously cooled trichloroacetic acid (10% *w*/*v*; 100 μL). The plates were then washed with water and, after drying, a sulforhodamine B solution (0.057% *w*/*v*, 100 μL) was added, and then left to stand at room temperature for 30 min. To remove non-adhered sulforhodamine B, the plates were washed three times with an acetic acid solution (1% *v*/*v*) and left to dry. Finally, the adhered sulforhodamine B was solubilized with Tris(hydroxymethyl)aminomethane (Tris) buffer (10 mM, 200 μL), and sample absorbance was measured at a wavelength of 540 nm, in a microplate reader (ELX800 Biotek microplate reader, Bio-Tek Instruments, Inc., Winooski, VT, USA). The results are expressed in terms of the sample concentration with the ability to inhibit cell growth by 50% (GI_50_).

#### 3.6.2. Anti-Inflammatory Activity

The anti-inflammatory activity of the developed formulations was also assessed. First, the formulations were diluted in water: DMSO solution to obtain a final concentration of 1 μg/mL, from which successive dilutions were carried out. Final concentrations ranged from 0.05 to 0.0008 mg/mL. A RAW 264.7 mouse macrophage cell line was used (Leibniz Institute DSMZ—German Collection of Microorganisms and Cell Cultures GmbH), and grown in DMEM medium, supplemented with heat-inactivated fetal serum (10%), glutamine and antibiotics, and kept in an incubator at 37 °C, with a 5% CO_2_ and highly humid atmosphere. Cells were detached with a cell scraper, and an aliquot of macrophages cell suspension (300 μL), with a cell density of 5 × 10^5^ cells/mL, and with a proportion of dead cells below 5% (according to the trypan blue exclusion test), was placed in each well. The microplate was incubated for 24 h, in an incubator, at the previously indicated conditions, to allow for adequate cell adherence and multiplication. After that period, the cells were treated with different concentrations of the developed formulations (15 μL) and incubated for one hour, with the range of tested concentrations being between 0.05 and 0.0008 μg/mL. Stimulation was performed with the addition of 30 μL of a liposaccharide (LPS) solution (1 mL/mL) and incubation for an additional 24 h. Dexamethasone (50 mM) was used as a positive control, and samples in the absence of LPS were used as a negative control. Quantification of nitric oxide was performed using a Griess reagent system kit (nitrophenamide, ethylenediamine, and nitrite solutions). The produced nitric oxide was determined by reading absorbances at 540 nm (ELX800 Biotek microplate reader, Bio-Tek Instruments, Inc., Winooski, VT, USA), on a 96-well microplate, and by comparison with a standard calibration curve. Results are depicted by a graphical representation of the percentage of inhibition of nitric oxide production versus sample concentration, and expressed in relation to the formulation concentration that causes the 50% inhibition of nitric oxide production (IC_50_).

### 3.7. Safety Assessment

#### 3.7.1. In Vitro Safety Assessment—Cytotoxicity Evaluation on HaCaT and HFF-1 Cell Lines

To assess the safety of the developed formulations in vitro, the colorimetric sulforhodamine B assay was conducted on two human cell lines: HFF-1 (human skin fibroblasts) and HaCaT (human immortalized keratinocytes). This assay measured cell survival after treatment with the developed formulations. The cell lines were cultured in DMEM medium, supplemented with fetal bovine serum (10%), glutamine, and antibiotics (penicillin and streptomycin 1%), in an incubator at 37 °C, with a 5% CO_2_ atmosphere, and with saturated controlled humidity. Trypsin was used to disperse the cells from the inside of the flask where they were cultured, since these cells are adherent. The cell density for this assay was 10,000 cells per well. X-triton (1%), a detergent, served as a positive control due to its capacity to disrupt and destroy all cells. For the negative control group, only cells and medium were added (no formulation or other compound).

After the cells were dispersed by trypsin in the culture medium, centrifuged at 3000 rpm for 5 min, and resuspended in the medium, 10,000 cells per well were plated in a 96-well optical-bottom plate to adhere overnight. Afterwards, the bee venom-loaded niosomes and empty niosomes (formulation vehicle) samples were prepared by diluting them in water, for final concentrations equal to 0.1, 0.05, 0.025, and 0.0125 mg/mL, which were added to the plate. Samples at the exact same concentrations were also prepared for the bee venom compound solution, also by dilution with water. Each concentration level was tested in triplicate. The plates were then incubated for 48 h at 37 °C and in a 5% CO_2_ atmosphere. After 48 h, the cells were fixated with trichloroacetic acid, for 1 h, at 4 °C. Afterwards, the liquid inside the plate was discarded, and the plate was washed 3 times with water, and left to dry. Once dried, 100 µL of sulforhodamine B was added to each well and left there for 30 min at room temperature. Acetic acid at 1% was used to remove the unbounded dye from the cells, and the bounded dye was dissolved with a 10 mM Tris buffer. The IC_50_ values were expressed as the concentration (µg/mL) of each formulation that caused 50% inhibition of cell growth. Samples were quantified by using UV-visible spectrophotometry in a microplate reader (ELX800 microplate reader, Bio-Tek Instruments, Winooski, VT, USA) at 540 nm, based on previously described methods [44,118].

#### 3.7.2. Ex Vivo Safety Assessment—HET-CAM Test

The hen’s egg chorioallantoic membrane (HET-CAM) test method was used to assess the irritation-inducing potential of the developed formulations, upon contact with a highly sensitive biological membrane, focusing on its ability to induce toxicity in the chorioallantoic membrane of a chicken egg. This type of membrane is known to resemble the human eye, and even if the product is not intended for ocular use, the test can still be quite useful since a formulation that is reasonably safe for eye application is probably also safe for contact with most other human organs [111].

The assay was done following the Interagency Coordinating Committee on the Validation of Alternative Methods (ICCVAM) recommended test method [119]. The experimental protocol consisted of the incubation of forty Leghorn fertilized chicken eggs, for 10 days, in an automatic rotating incubator, at 37 °C and 65% humidity. To confirm the presence of an embryo, a flashlight was used against each egg. If no embryo was detected that egg would be excluded from the experiment, and not used. For each tested formulation, a total of three eggs were used. After the 10-day incubation period, the top part of the eggshells was cut open to expose the chorioallantoic membrane, so that the formulation could then be applied. Nevertheless, before formulation application, all the membranes were hydrated using a 0.9% NaCl solution, for 30 min. Then, three formulation drops (approximately 0.1 mL per drop, for a total of 0.3 mL of formulation) were applied to the chorioallantoic membrane of each egg [119]. The negative control group was three eggs where a 0.9% NaCl solution was applied (no reaction intended), while for the positive control group, a 10% NaOH solution was applied on the membrane of three eggs (inflammatory reaction intended). The irritancy degree of each formulation was observed and monitored by the appearance of the following events: hemorrhage (bleeding of the vessels), lysis (disintegration of the vessels), and coagulation (intra and/or extra-vascular protein denaturation). The occurrence or non-occurrence of these events was observed at specific time points, namely 0.5, 2, and 5 min. A total score was then attributed, from 0 to 21, being the sum of the values attributed to each event and corresponding to a level of irritability (Table 7). Hence, after the 5 min time interval, formulations were given an irritation score (IS), with the following corresponding irritation levels: an IS score between 0 and 4.9 being slightly/non-irritative; an IS score between 5 and 8.9 being moderately irritative; and an IS score between 9 and 21 being strongly irritative [119,120].

### 3.8. Data Analysis

For a better understanding and interpretation of the results, whenever possible, statistical analysis was performed, using GraphPad Prism^®^ (GraphPad, San Diego, CA, USA), version 8.0 software. More specifically, either a one-way ANOVA or a two-way ANOVA test was applied, with a Tukey’s multiple comparisons post-test. A 95% confidence level was considered in all analysis, with a *p* value < 0.05 being considered statistically significant.

## 4. Conclusions

An innovative nanotechnological anticancer platform was successfully developed. The developed bee venom-loaded niosomes revealed small nanometric and homogeneous particle size, therapeutic efficacy in several in vitro cancer models, including breast, cervical, lung, gastric, and colorectal cell lines, and complementary anti-inflammatory potential, which has been proven to indirectly contribute to further synergistic anticancer effects. The developed formulations were shown to improve bee venom administration safety, in in vitro and ex vivo models. Although further studies will clarify the true potential of the developed bee venom-loaded niosomes, these findings are promising regarding both formulation safety and bioactivity, making them potentially promising alternatives for primary or adjuvant cancer treatment.

## Figures and Tables

**Table 1:** Chemical characterization of the bee venom encapsulated within the developed niosomes, or solubilized within the produced compound solution, performed by UHPLC analysis. Major compounds are presented, with values corresponding to the amount present in each analyzed sample (µg/mL).

Compounds	Bee Venom Solution (300 µg/mL)	Bee Venom-Loaded Niosomes (2 µg/mL)	Correspondent Percentage (%)
Melittin	193.43	1.29	63
Phospholipase A2	37.52	0.25	12
Apamin	3.61	0.024	1.2

UHPLC—ultra-high-performance liquid chromatography.

**Table 2:** Particle size (nm) and PDI of empty and bee venom-loaded niosomes, after optimization (31 cycles of extrusion).

Formulations	Bee Venom Concentration	Particle Size (nm)	PDI
Empty niosomes (formulation vehicle)	0	151.2 ± 1.8	0.091 ± 0.018
Bee venom-loaded niosomes	2 µg/mL	150.4 ± 3.7	0.162 ± 0.008

PDI—polydispersity index.

**Table 3:** Significance matrix of the HaCaT and HFF-1 cell viability assay results (depicted in Figure 6), comparing the viability percentage variation with concentration variation within each studied formulation, for each cell type. The presented values correspond to *p* values, after the application of one-way ANOVA with Tukey’s multiple comparisons’ test.

HFF-1 Cells						HaCaT Cells					
Bee Venom-Loaded Niosomes						Bee Venom-Loaded Niosomes					
Concentration (µg/mL)	0	25	50	100	200	Concentration (µg/mL)	0	25	50	100	200
Overall	<0.0001					Overall	<0.0001				
0	-	NS	NS	0.0352	<0.0001	0	-	0.0025	<0.0001	<0.0001	<0.0001
25	-	-	NS	NS	<0.0001	25	-	-	NS	<0.0001	<0.0001
50	-	-	-	NS	<0.0001	50	-	-	-	0.0002	<0.0001
100	-	-	-	-	<0.0001	100	-	-	-	-	<0.0001
200	-	-	-	-	-	200	-	-	-	-	-
Empty niosomes						Empty niosomes					
Concentration (µg/mL)	0	25	50	100	200	Concentration (µg/mL)	0	25	50	100	200
Overall	<0.0001					Overall	<0.0001				
0	-	NS	<0.0001	<0.0001	<0.0001	0	-	NS	NS	<0.0001	<0.0001
25	-	-	<0.0001	<0.0001	<0.0001	25	-	-	NS	<0.0001	<0.0001
50	-	-	-	<0.0001	<0.0001	50	-	-	-	<0.0001	<0.0001
100	-	-	-	-	0.0392	100	-	-	-	-	<0.0001
200	-	-	-	-	-	200	-	-	-	-	-
Bee venom solution						Bee venom solution					
Concentration (µg/mL)	0	25	50	100	200	Concentration (µg/mL)	0	25	50	100	200
Overall	<0.0001					Overall	<0.0001				
0	-	NS	NS	0.0003	<0.0001	0	-	NS	NS	<0.0001	<0.0001
25	-	-	NS	0.0002	<0.0001	25	-	-	NS	<0.0001	<0.0001
50	-	-	-	0.0021	<0.0001	50	-	-	-	<0.0001	<0.0001
100	-	-	-	-	0.0087	100	-	-	-	-	<0.0001
200	-	-	-	-	-	200	-	-	-	-	-

NS—not significant (statistical significance); HaCaT—skin keratinocytes cell line; and HFF-1—skin fibroblasts cell line.

**Table 4:** Significance matrix of the HaCaT and HFF-1 cell viability assay results (depicted in Figure 6), comparing the viability percentage variation between studied formulations, for the same concentrations, for each cell type. The presented values correspond to *p* values, after application of two-way ANOVA with Tukey’s multiple comparisons’ test.

HFF-1 Cells						HaCaT Cells					
Bee Venom-Loaded Niosomes vs. Empty Niosomes						Bee Venom-Loaded Niosomes vs. Empty Niosomes					
Overall	0 µg/mL	25 µg/mL	50 µg/mL	100 µg/mL	200 µg/mL	Overall	0 µg/mL	25 µg/mL	50 µg/mL	100 µg/mL	200 µg/mL
<0.0001	0.0037	0.0104	0.0025	<0.0001	<0.0001	<0.0001	0.0058	<0.0001	0.0015	NS	<0.0001
Bee venom-loaded niosomes vs. bee venom solution						Bee venom-loaded niosomes vs. bee venom solution					
Overall	0 µg/mL	25 µg/mL	50 µg/mL	100 µg/mL	200 µg/mL	Overall	0 µg/mL	25 µg/mL	50 µg/mL	100 µg/mL	200 µg/mL
<0.0001	<0.0001	<0.0001	<0.0001	<0.0001	<0.0001	<0.0001	<0.0001	<0.0001	<0.0001	<0.0001	<0.0001
Empty niosomes vs. bee venom solution						Empty niosomes vs. bee venom solution					
Overall	0 µg/mL	25 µg/mL	50 µg/mL	100 µg/mL	200 g/mL	Overall	0 µg/mL	25 µg/mL	50 µg/mL	100 µg/mL	200 µg/mL
<0.0001	<0.0001	<0.0001	<0.0001	<0.0001	<0.0001	<0.0001	0.0004	0.0001	<0.0001	<0.0001	<0.0001

NS—not significant (statistical significance); HaCaT—skin keratinocytes cell line; and HFF-1—skin fibroblasts cell line.

**Table 5:** Cell viability of the developed bee venom-loaded niosomes, formulation vehicle (empty niosomes), and compound solution (bee venom solution), evaluated in HaCaT and HFF-1 cell lines, and represented by the calculated IC_50_ value.

	Cell Viability (IC_50_, µg/mL)	
	HFF-1 Cells	HaCaT Cells
Bee venom-loaded niosomes	165.7 ± 1.0	161.0 ± 1.0
Empty niosomes	138.2 ± 1.0	69.8 ± 1.00
Bee venom solution	>200.0	>200.0

IC_50_ value corresponds to formulation concentrations causing 50% reduction of cell viability or proliferation; data is represented as mean ± standard deviation of tested formulations; HaCaT—skin keratinocytes cell line; and HFF-1—skin fibroblasts cell line.

**Table 6:** HET-CAM results after application of the developed bee venom-loaded niosomes or formulation vehicle (empty niosomes), with respective positive (0.1 N NaOH) and negative (0.9% NaCl) controls, on hen’s egg chorioallantoic membranes, and represented by the calculated average irritation score (IS) ± standard deviation, and visible vascular reactions and overall irritation degree.

Formulation	Average IS	Standard Deviation	Vascular Reactions Following Treatment	Irritation Degree
Bee venom-loaded niosomes	7.7	0.47	No lysis	Slightly irritative
Empty niosomes	14	0	Hemorrhage	Strongly irritative
0.9% NaCl	0.00	-	None	Non-irritant
0.1 N NaOH	19.00	-	Lysis and hemorrhage	Strongly irritative

IS—irritation score; HET-CAM—hen’s egg chorioallantoic membrane.

**Table 7:** Numerical time-dependent scoring scheme for the HET-CAM test method.

Effect	Score		
	0.5 min	2 min	5 min
Lysis	5	3	1
Hemorrhage	7	5	3
Coagulation	9	7	5

HET-CAM—hen’s egg chorioallantoic membrane.

## Data Availability

Data are contained within the article.

## Abbreviations

COX-2—cyclooxygenase-2; DMEM—Dulbecco’s modified Eagle’s medium; DMSO—dimethyl sulfoxide; DSMZ—German Collection of Microorganisms and Cell Cultures; ECCAC—European Collection of Authenticated Cell Cultures; IC_50_—formulation concentration that causes a 50% inhibition of nitric oxide production/cell growth; IS—internal standard; GCCP—Guidance on Good Cell Culture Practice; GI_50_—sample concentration with the ability to inhibit cell growth by 50%; HET-CAM—hen’s egg chorioallantoic membrane; ICCVAM—Interagency Coordinating Committee on the Validation of Alternative Methods; IFN—interferons; IgE—immunoglobulin E; IL—interleukins; IS—irritation score; LPS—liposaccharide; PDI—polydispersity index; TNF—tumor necrosis factor; Tris—Tris(hydroxymethyl)aminomethane; and UHPLC—ultra-high-performance liquid chromatography.
